# Review of the genus *Tinissa* Walker, 1864 (Lepidoptera, Tineidae, Scardiinae) from China, with description of five new species

**DOI:** 10.3897/zookeys.228.3645

**Published:** 2012-10-09

**Authors:** Linlin Yang, Houhun Li

**Affiliations:** 1 College of Life Sciences, Nankai University, Tianjin 300071, China

**Keywords:** Lepidoptera, Tineidae, *Tinissa*, new species, China

## Abstract

The genus *Tinissa* Walker is reviewed for China. Seven species are recognized, of which *Tinissa apicimaculata* **sp. n.**, *Tinissa conchata* **sp. n.**, *Tinissa connata* **sp. n.**,*Tinissa leguminella* **sp. n.** and *Tinissa spirella* **sp. n.** are described as new; and *Tinissa insularia* Robinson, 1976 is newly recorded from China. Photographs of the adults and illustrations of the genitalia are given. A key to all the known Chinese species and a distribution map of *Tinissa* in China are included.

## Introduction

The genus *Tinissa* was established by Walker (1864) with *Tinissa torvella* Walker, 1864 as the type species. It was once included in the subfamily Tinissinae, which was established by Gozmány and Vári (1973) for *Tinissa* and *Leptozancla* Meyrick, 1920. Robinson (1976) revised the Tinissinae on a worldwide basis, including 32 *Tinissa* species. Subsequently, Robinson (1981) described two more species from New Guinea and Borneo, and proposed a phylogenetic classification for *Tinissa*. Robinson (1986) synonymized Tinissinae with the subfamily Scardiinae. Following this treatment, Robinson and Tuck (1998) described one species from Brunei; and Robinson (2008) elevated the subspecies *Tinissa torvella mysorensis* Robinson, 1976 to species level. Currently, *Tinissa* comprises 36 named species, 14 of which are described from the Australian Region, 17 from the Oriental Region and five from the Afrotropical Region (Meyrick 1910, 1916, 1917, 1926, 1927, 1928, 1932; Gozmány and Vári 1973; Robinson 1976, 1981, 2008; Zagulajev 1972; Robinson and Tuck 1998).

**Figure 1.**
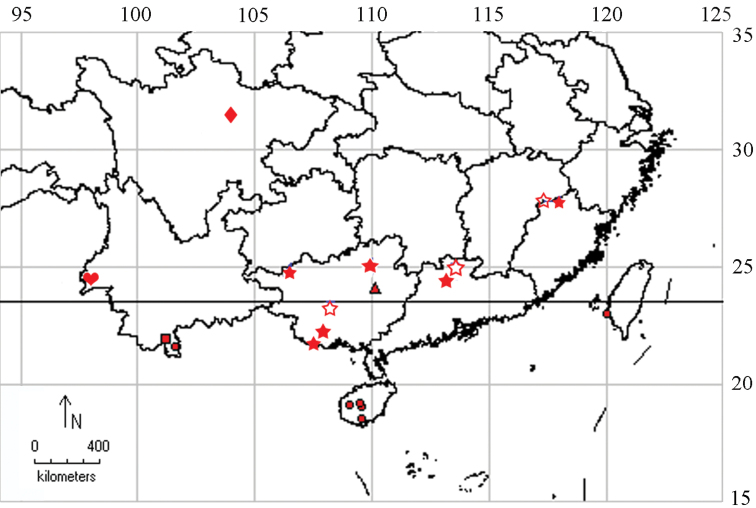
Distribution of Chinese *Tinissa* spp. ● *Tinissa indica* Robinson, 1976 ■ *Tinissa insularia* Robinson, 1976 ▲ *Tinissa apicimaculata* sp. n. ★ *Tinissa conchata* sp. n. ☆ *Tinissa leguminella* sp. n. ♥ *Tinissa connata* sp. n. ♦ *Tinissa spirella* sp. n.

Prior to this study, *Tinissa indica* Robinson, 1976 was recorded in Taiwan (Robinson 1976), but none were recorded from Mainland China. The aim of the present paper is to review *Tinissa* in China, describe five new species, and record one species as new for the Chinese fauna. The distribution of *Tinissa* in China is shown by map (Fig. 1).

## Material and methods

The specimens examined were collected using light traps. Morphological terms in the descriptions follow Robinson (1976). Whole body dissections were carried out following the methods described by Lee and Brown (2006), and genitalia dissection and mounting methods follow Li (2002). Photographs of adults were taken with a Nikon D300, and the genitalia were photographed with an Olympus C7070WZ digital camera. All the studied specimens, including the types, are deposited in the Insect Collection, College of Life Sciences, Nankai University, Tianjin, China.

### Taxonomic accounts

#### Tinissa

Walker, 1864

http://species-id.net/wiki/Tinissa

Tinissa Walker, 1864: 780. Type-species: *Tinissa torvella* Walker, 1864: 780, by monotypy.
Polymnestra Meyrick, 1927: 331. Type species: *Polymnestra perilithas* Meyrick, 1927: 331, by monotypy. [Synonymized by Gozmány and Vári 1973: 85.]

##### Generic characters.

Medium to large tineid moths.

**Figures 2−3. F2:**
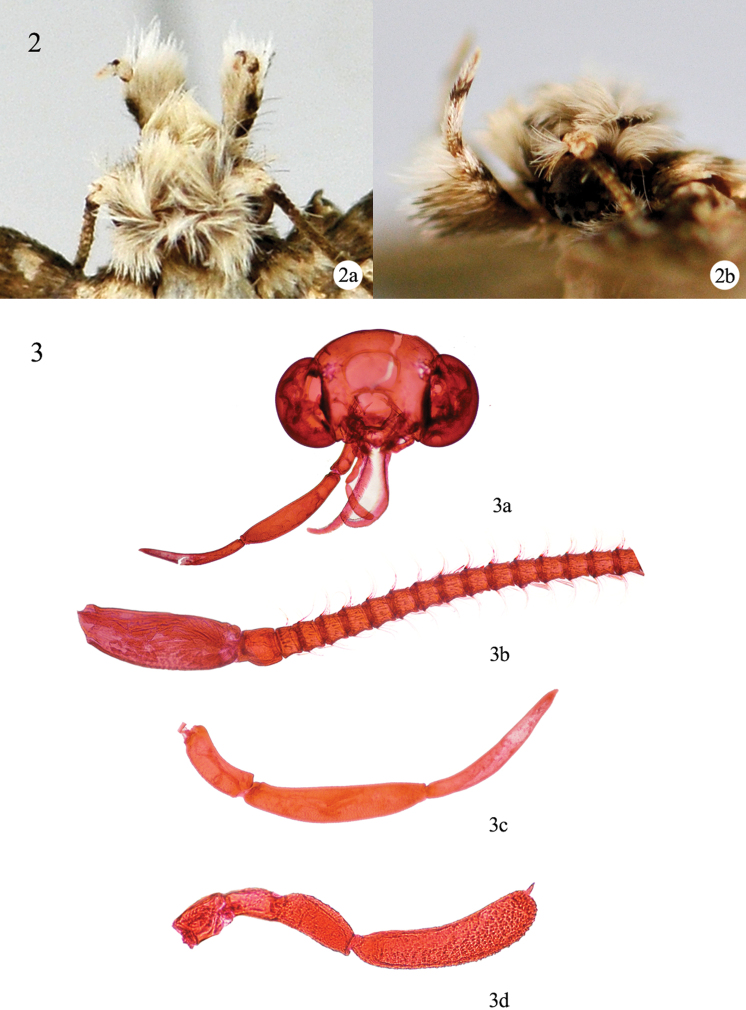
Head structure of *Tinissa indica* Robinson, ♂. **2** Head with scales: **2a** dorsal view **2b** lateral view **3** Head scales removed: **3a** head, slide No. NKYLL012 **3b** antenna **3c** labial palpus **3d** maxillary palpus.

**Head** (Figs 2−3): Vertex and frons white of various shade, usually mixed with grayish brown scales (Fig. 2a). Compound eye large (Fig. 3a). Antenna about 0.5× length of forewing; scape with pecten more than 10 bristles; flagellum with elongate cilia (Fig. 3b). Labial palpus segmental ratio 1:2:2 (Fig. 3c), second segment with a few bristles and dense tuft, third segment upturned to about 60° (Fig. 2b). Maxillary palpus (Fig. 3d) with three to five segments.

**Figures 4−5. F3:**
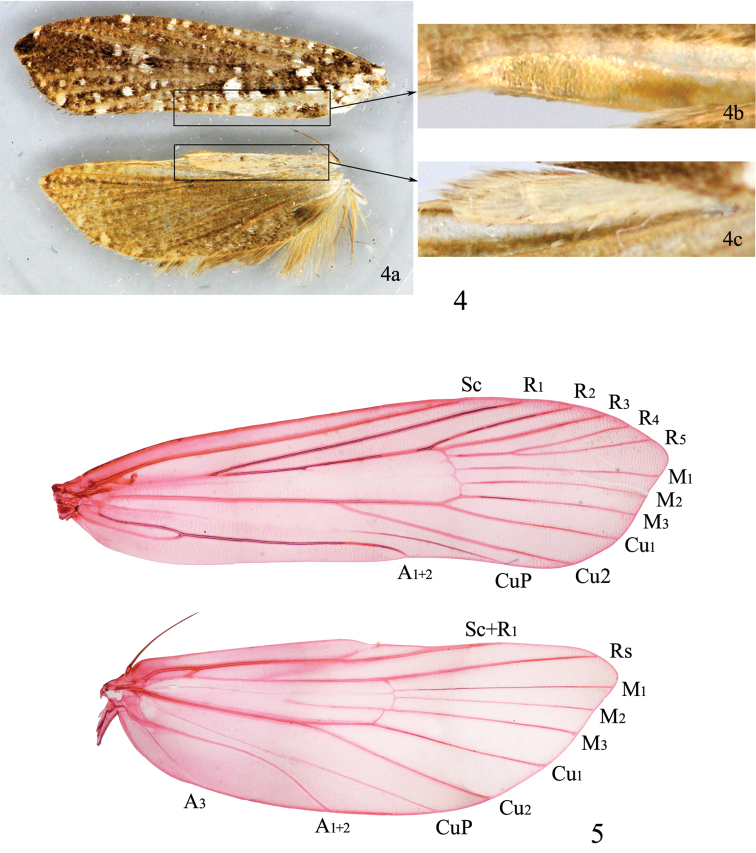
Wings of *Tinissa indica* Robinson, ♂. **4** Wings and patches: **4a** wings **4b** oval patch **4c** rough scale patch **5** Venation, slide No. NKYLL012.

**Forewing** (Figs 4−5): Index 0.25−0.30, somewhat rectangular, with termen slightly concave inward, or lanceovate, with termen obliquely blunt; color brown in general, with a purplish sheen, with scattered white dots (Fig. 4a); M absent or with weak trace in cell, R_5_ to costa or apex or just on to termen (R_5_ to costa near apex in all the seven Chinese species), R_4_ and R_5_ separate, stalked or completely fused (Fig. 5); with elongate oval patch of small, flat, ovate scales on ventral surface between A_1+2_ and dorsum (Fig. 4b). Hindwing (Figs 4−5) index 0.3−0.35, costa with distal half slightly concave; grayish brown, shining purplish; with a patch of rough, pale scales opposite forewing patch, anterior to Sc+R_1_ (Fig. 4c); all veins present (Fig. 5), M stem usually present, branched or not, weak or conspicuous. Male frenulum with one slender bristle, female frenulum with two or three bristles (female frenulum with two bristles in all Chinese species). Legs yellowish white to white; mid tibia usually with a brown spot at base and two oblique brown bands on outer surface; hind leg elongate, tibia bearing large tufts of erect scales at apex.

**Figures 6−7.**
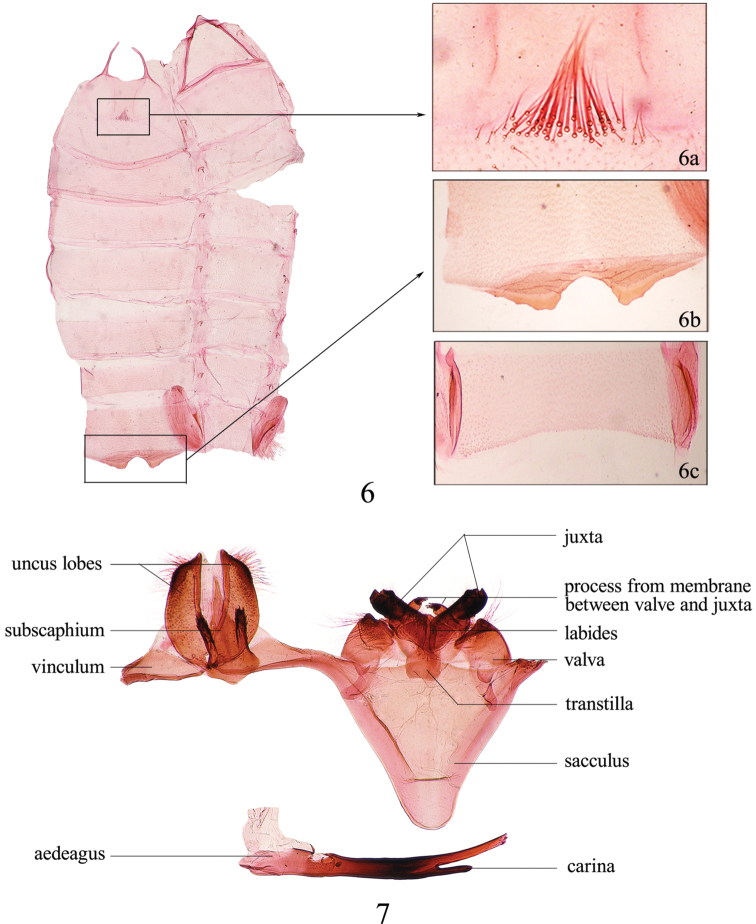
Male abdomen of *Tinissa indica* Robinson. **6** Pregenital abdomen: **6a** setae on second sternite **6b−6c** eighth sternite **7** male genitalia (**6a−b, 7** slide No. NKYLL012; **6c** *Tinissa connata* sp. n., slide No. YLL11167).

**Abdomen** (Fig. 6): Second sternite elongate, twice length of other sternites, with sclerotized eyepatch-shaped trace on anterior half, with small tuft of forward-directed setae medially (Fig. 6a); corema present or absent in eighth segment in male, eighth sternite with (Fig. 6b) or without (Fig. 6c) processes; corethrogyne present or absent in seventh segment in female. Male genitalia (Fig. 7) with tegumen and gnathos absent; uncus bilobate, being a pair of lobes of highly interspecific diversity attached to vinculum by membrane or fused with vinculum; subscaphium elongate and conspicuous, diagnostic at species level; saccus broad and triangular, or slightly narrow and rodlike; juxta large, closely appressed to valva, diversely modified; valva usually short and conical; a pair of variously shaped processes arising from membrane between valva and juxta; labides present, dorsal to aedeagus, usually a pair of lobes, sometimes fused. Aedeagus of various shape and size, with or without carina, cornuti absent. Female genitalia with variously shaped eighth sternite, usually ventrally protuberant; ostium similarly diverse; antrum usuallypresent, often divided by a narrow, oblique membranous ring at point of junction with ductus seminalis; ductus bursae usually with transverse, regular constrictions. Corpus bursae with or without signum.

##### Diagnosis.

Members of *Tinissa* are medium to large sized robust tineid moths that can be easily recognized by having an elongate, brown-colored forewing with purple sheen and patterned with faint white dots; the hind legs are elongate and bear large tufts of erect scales at tibia apex; the male genitalia are characterized by the bilobate uncus, and the enlarged juxta being a pair of processes, forming a complex with the valva. *Tinissa* shares the above characters with *Leptozancla*, but differs from *Leptozancla* by the antenna about 0.5× length of forewing, the forewing with elongate oval patch of small, flat, ovate scales, the hindwing with patch of rough, pale scales opposite to the forewing patch; the male genitalia with reduced valva, and the labides present as a pair of lobes. In *Leptozancla*, the antenna is as long as the forewing, the forewing lacks the ovate patch, the hindwing lacks the patch opposite to the forewing patch, the valva is absent, the labides are present as elongate, posteriorly directed spines in the male genitalia.

##### Biology.

One species was reared from fungus on bamboo (Robinson 1976).

##### Distribution.

Afrotropical, Oriental and Australian regions, as far east as the Solomon Islands, and as far south as Queensland (Robinson 1986).

##### Remarks.

Robinson (1976) described that the maxillary palpus has three or five segments. Our whole body dissection of *Tinissa indica* shows that the maxillary palpus has four segments in this species (Fig. 3d).

### Key to Chinese *Tinissa* species based on males

(Excluding *Tinissa apicimaculata* sp. n. of which the male is unknown)

**Table:** 

1	Wingspan > 15.0 mm; first hind tarsal segment with smooth scales	2
–	Wingspan < 15.0 mm; first hind tarsal segment with rough, raised scales (Fig. 11)	*Tinissa connata* sp. n.
2	Saccus broad, triangular; transtilla present; aedeagus stout, with carina	3
–	Saccus elongate, rodlike; transtilla absent; aedeagus slender, without carina	5
3	Forewing scattered with conspicuous white spots throughout, arranged regularly along margins as well as between veins; transtilla inverted peach-shaped; aedeagus complete dorsally and ventrally	4
–	Forewing diffused with irregular ochreous white dots forming indistinct transverse striae (Fig. 8); transtilla being two small, sclerotized plates; aedeagus dorsally complete, ventrally with narrow slit from apex to middle (Fig. 14)	*Tinissa insularia*
4	Uncus lobe crescent; subscaphium triangular; juxta fist-shaped (Fig. 7)	*Tinissa indica*
–	Uncus lobe beanpod-shaped; subscaphium bifurcate posteriorly; juxta horn-shaped (Fig. 17)	*Tinissa leguminella*sp. n.
5	Uncus lobe ovate, with shallow pocket posterolaterally; subscaphium clubbed, with elongate setae posteriorly; juxta rectangular in basal 2/3, scallop-shaped in distal 1/3; valva flask-shaped; aedeagus clubbed, straight, with a row of small spinules (Fig. 15)	*Tinissa conchata* sp. n.
–	Uncus lobe L-shaped, with slender hornlike thorn at apex; subscaphium bulletlike, with slender fingerlike process on each side; juxta spiral; valva pyramidical; aedeagus needlelike, curved dorsad, without spinule (Fig. 18)	*Tinissa spirella* sp. n.

### Key to Chinese *Tinissa* species based on females

(Excluding *Tinissa leguminella* sp. n. and *Tinissa spirella* sp. n., of which the females are unknown)

**Table:** 

1	Wingspan > 15.0 mm; first hind tarsal segment with rough, raised scales	2
–	Wingspan < 15.0 mm; first hind tarsal segment with smooth scales	3
2	Forewing with an ovate, large, blackish brown spot near apex (Fig. 9); corethrogyne present	*Tinissa apicimaculata* sp. n.
–	Forewing without spot near apex (Fig. 11); corethrogyne absent	*Tinissa connata* sp. n.
3	Posterior margin of eighth tergite deeply concave at middle	4
–	Posterior margin of eighth tergite slightly sinuate (Fig. 19)	*Tinissa indica*
4	Antrum almost as long as sternite, junction with ductus seminalis at 2/3; ductus bursae with conspicuously coarse transverse constrictions (Fig. 21)	*Tinissa conchata* sp. n.
–	Antrum shorter than sternite, junction with ductus seminalis at one-half, ductus bursae with very fine transverse constrictions (Robinson 1976: Fig. 87)	*Tinissa insularia*

#### Tinissa indica

Robinson, 1976

http://species-id.net/wiki/Tinissa_indica

Figs 1 [Fig F2] [Fig F3] −7 , 19

Tinissa indica Robinson, 1976: 282.

##### Material examined.

**CHINA, Hainan Province:** 1 ♀, Mt. Diaoluo (18°28'N, 109°31'E), 940 m, 31.v.2007, leg. Zhiwei Zhang and Weichun Li, genitalia slide No. ZL09028; 1 ♂, Mt. Yingge (19°01'N, 109°33'E), 30.ix.2011, leg. Bingbing Hu, genitalia slide No. YLL11135m, YLL11135w; 1 ♂, Nancha River, Mt. Bawang (19°04'N, 109°02'E), 600 m, 9.vi.2007, leg. Zhiwei Zhang and Weichun Li; 2 ♂♂, East first Protection Station, Mt. Bawang, 650 m, 7.iv.2008, leg. Bingbing Hu and Haiyan Bai, genitalia slide No. NKYLL012; 1 ♂, East Administration, Mt. Bawang, 8.v.2011, leg. Dandan Zhang and Lifeng Yang; 1 ♂, Mt. Wuzhi (18°46'N, 109°30'E), 650 m, 15.v.2007, leg. Zhiwei Zhang and Weichun Li; 2 ♂♂, Mt. Limu (19°09'N, 109°28'E), 5.v.2011, leg. Dandan Zhang and Lijun Yang. **Yunnan Province:** 1 ♂, Bubang (21°36'N, 101°35'E), Mengla, 650 m, 25.vii.2008, leg. Yingdang Ren, genitalia slide No. XYL05049.

##### Diagnosis.

Adult (Figs 2−5) with wingspan 24.0−28.0 mm in male, 30.0 mm in female. *Tinissa indica* can be easily recognized from its congeners by the male genitalia having a pair of crescent-shaped uncus lobes, the triangular subscaphium with wide, fingerlike process posterolaterally, and the fist-shaped juxta (Figs 6−7), and by the female genitalia having the eighth sternite ventrally protuberant and the hemispherical antrum (Fig. 19).

##### Distribution.

China (Hainan, Yunnan, Taiwan), India, Sikkim, Bhutan.

#### Tinissa insularia

Robinson, 1976

http://species-id.net/wiki/Tinissa_insularia

Figs 1 , 8 , 14

Tinissa insularia Robinson, 1976: 285; Robinson, 1981: 371.

##### Material examined.

**CHINA**, **Yunnan Province:** 1 ♂, Baka Village, Menglun Town (21°56'N, 101°15E), Mengla County, 620 m, 6.VIII.2010, leg. Yinghui Sun and Lixia Li, genitalia slide No. YLL11138.

##### Diagnosis.

**Figures 8−13.**
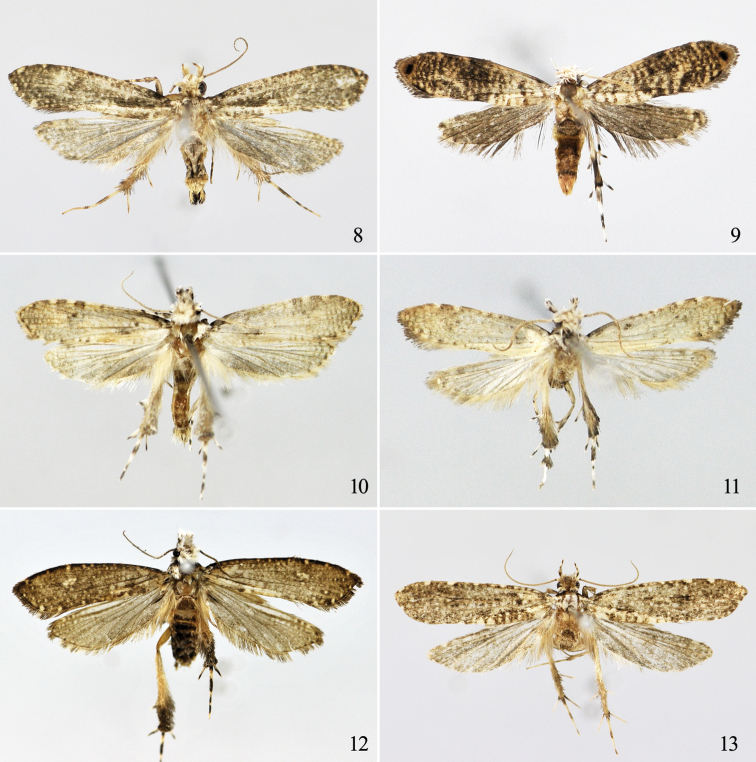
Adults of *Tinissa* spp. **8** *Tinissa insularia* Robinson, male **9** *Tinissa apicimaculata* sp. n., holotype, female **10** *Tinissa conchata* sp. n., holotype, male **11** *Tinissa connata* sp. n., holotype, male **12** *Tinissa leguminella* sp. n., holotype, male **13** *Tinissa spirella* sp. n., holotype, male.

Adult (Fig. 8) with male wingspan 17.0 mm. *Tinissa insularia* is close to *Tinissa spirella* sp. n., but differs as noted in the description of the new species.

**Figures 14−18.**
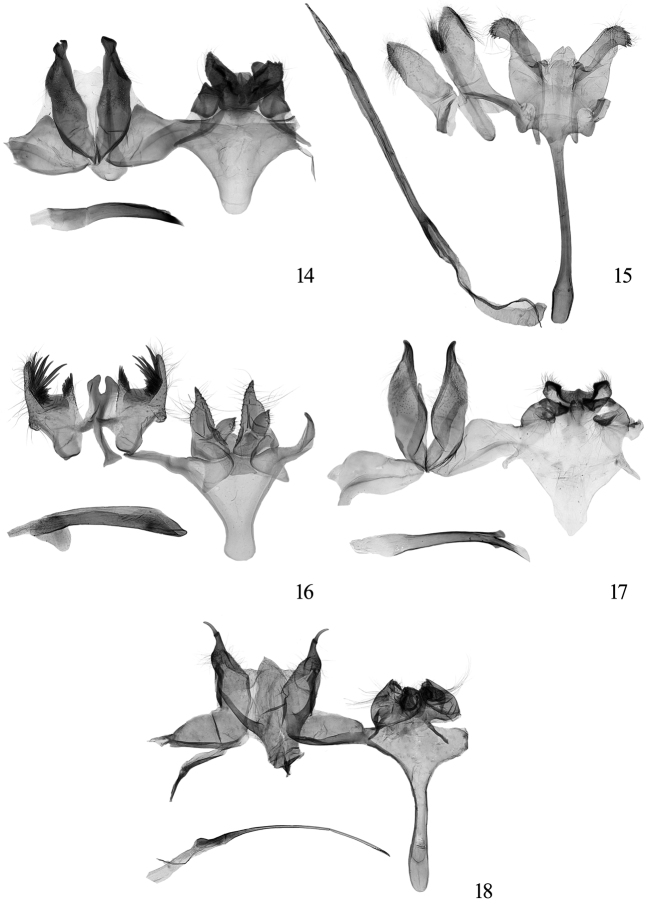
Male genitalia of *Tinissa* spp. **14** *Tinissa insularia* Robinson, slide No. YLL11138 **15** *Tinissa conchata* sp. n., paratype, slide No. YLL10196 **16** *Tinissa connata* sp. n., paratype, slide No. YLL11167 **17**  *Tinissa leguminella* sp. n., paratype, slide No. XYL05048 **18** *Tinissa spirella* sp. n., holotype, slide No. XYL05050.

##### Distribution.

China (Yunnan), Malaysia, Borneo, Indonesia (Sumatra, Java, Celebes, Moluccas), Philippines (Luzon, Mindanao, Palawan, Balabac, Tawi Tawi), New Guinea (Papua, Karkar I., New Britain), Solomon Is.

##### Notes.

This species is recorded for the first time in China.

#### Tinissa apicimaculata sp. n.

urn:lsid:zoobank.org:act:DA24EC3C-3D69-4107-8D07-79A9F1B8841A

http://species-id.net/wiki/Tinissa_apicimaculata

Figs 1 , 9 , 20

##### Type material.

Holotype ♀ − **CHINA, Guangxi Zhuang Autonomous Region:** Jinxiu County (24°08'N, 110°11'E), 550 m, 15.IV.2002, leg. Shulian Hao and Huaijun Xue, genitalia slide No. YLL11136.

##### Diagnosis.

The new species is a distinctive species: the forewing markings are diagnostic, with an ovate, large, blackish brown spot near apex. It is small-sized as *Tinissa connata* sp. n., but can be easily recognized by the superficial characters.

##### Description.

Adult (Fig. 9): Wingspan 12.0 mm in female. Vertex and frons creamy white. Antenna with scape and pecten white, pecten about 10−20 bristles; flagellum yellowish white. Labial palpus creamy white, second segment and tuft yellowish brown on outer surface. Thorax and tegula creamy white, anterior margin dark brown. Forewing index 0.27, lanceovate, apex protruded roundly, termen oblique; ground color creamy white on basal 1/3, yellowish brown on distal 2/3, gradually darker to apex, shining copperish violet; patterned with indistinct, faint transverse striae, with large, blackish brown ovate spot near apex; M absent in cell, R_4_ and R_5_ separated; fringe brown, pale yellowish brown at middle, forming a line parallel with pale yellowish brown basal line. Hindwing index 0.3; grayish brown, yellowish brown near apex, shining copperish violet; M stem conspicuous, not branched; fringe yellowish brown. Legs dominantly creamy white; fore femur with narrow, blackish brown spot on ventral surface, tibia blackish brown on outer surface, tarsus with fifth segment dark brown; mid tibia distally with oblique dark brown band on outer surface, spurs with oblique black band before apex, tarsus with middle portion of first and second segments dark brown, lighter on inner surface; hind tibial tuft creamy white on basal half, blackish brown on distal half, spurs with oblique blackish bands before apex, tarsus with basal half of first segment grayish brown, with rough, raised scales on dorsal surface, second and third segments blackish brown.

**Male.** Unknown.

**Female genitalia** (Fig. 20). Corethrogyne present in seventh segment. Eighth tergite rectangular, with sparse long setae on posterior margin; sternite hemicyclic, ventrally protuberant in short, tubular shape, with a pair of small, setose, mastoid processes at middle on posterior margin. Apophysis anterior 0.5× length of apophysis posterior. Ostium situated at middle of eighth sternite on posterior margin. Antrum absent; ductus bursae 3.5× length of corpus bursae, posterior 1/4 narrow, anterior 3/4 broader, 2.0× width of posterior 1/4. Corpus bursae ovate, without signum.

**Figures 19−22.**
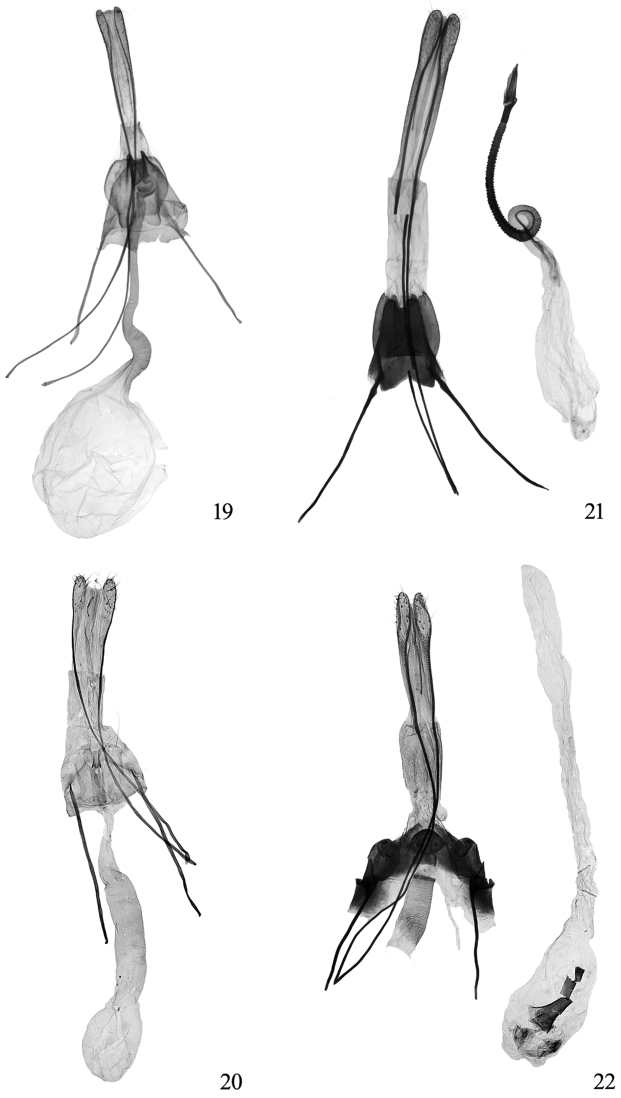
Female genitalia of *Tinissa* spp. **19** *Tinissa indica* Robinson, slide No. ZL09028 **20** *Tinissa apicimaculata* sp. n., holotype, slide No.YLL11136 **21** *Tinissa conchata* sp. n., paratype, slide No. YLL11140 **22** *Tinissa connata* sp. n., paratype, slide No.YLL11137.

##### Distribution.

China (Guangxi).

##### Etymology.

The specific name is derived from the Latin prefix *apici*- (= apex) and *maculatus* (= macula), referring to the ovate, blackish brown spot near apex of forewing.

##### Remarks.

In Tineidae, new species are usually described on the basis of male specimens. However, *Tinissa* is an exception. Among the 36 nominal species, seven are based on both male and female, 14 on male and 15 on female only. As there is no sexual dimorphism in this genus, *Tinissa apicimaculata* sp. n. is distinctive from other species – most likely also in the male – by the forewing having an ovate blackish spot near apex.

#### Tinissa conchata sp. n.

urn:lsid:zoobank.org:act:1A49EDE3-1411-45DB-99AA-EF22FBA1201E

http://species-id.net/wiki/Tinissa_conchata

Figs 1 , 10 , 15 , 21

##### Type material.

Holotype ♂ − **CHINA**, **Fujian Province:** Mt. Wuyi (27°45'N, 118°02'E), 740 m, 25.vii.2008, leg. Weichun Li, Yongling Sun and Haiyan Bai. Paratypes:2 ♂♂, same data as holotype except dated 27.vii.2008. **Guangdong Province:**1 ♂, Nanling Mt. Babao (24°24'N, 113°08'E), 1070 m, 23.viii.2010, leg. Shulian Hao, genitalia slide No. YLL10196. **Guangxi Zhuang Autonomous Region:** 1 ♂, Qinmucun, Yongfu County (24°58'N, 109°58'E), 160 m, 1.v.2008, leg. Hui Zhen and Li Zhang; 1 ♂, Yachang Forest, Leye County (24°47'N, 106°33'E), 910 m, 28.vii.2004, leg. Jiasheng Xu, genitalia slide No. MYH11089; 1 ♀, Dongzhong Forest (21°37'N, 107°32'E), Fangchenggang City, 370 m, 9.iv.2002, leg. Shulian Hao and Huaijun Xue, genitalia slide No. YLL11140; 1 ♂, Hongqi Forest, Shangsi County (22°09'N, 107°59'E), 260 m, 2.IV.2002, leg. Shulian Hao and Huaijun Xue.

##### Diagnosis.

The new species is similar to *Tinissa amboinensis* Robinson, 1976, *Tinissa cinerascens* Meyrick, 1910 and *Tinissa distracta* Meyrick, 1916 in the forewing having fine transverse striae; the ovate uncus lobe with shallow pocket posterolaterally, the clubbed subscaphium setose posteriorly, the rodlike saccus expanded anteriorly, and the slender aedeagus in malegenitalia; and the antrum divided by a membranous ring at point of junction with ductus seminalis in the female genitalia. However, the shapes of the valva, the juxta and the aedeagus in the male genitalia are different among the four species: in the new species, the valva is flask-shaped, the juxta is rectangular basally and scallop-shaped distally, with dentation on posterior margin, and the aedeagus has small spinules; in *Tinissa amboinensis*, the valva is triangular, the juxta is roundly lobe-shaped, with smooth margins, the aedeagus lacks the carina or spinule (Robinson 1976, Fig. 46); in *Tinissa cinerascens*, the valva is triangular, the juxta is somewhat triangular, with smooth margins, the aedeagus lacks carina or spinule (Robinson 1976, Fig. 48); in *Tinissa distracta*, the valva is triangular, the juxta has two small conical projections on inner surface, the aedeagus bears two small carinae before apex (Robinson 1976, Fig. 45). Besides, the structures of the eighth segment, the position of the membranous ring on the antrum and the shape of the antrum in the female genitalia are also different among the four species.

##### Description.

Adult (Fig. 10): Wingspan 16.0−20.0 mm in male, 22.0 mm in female. Vertex yellowish white; frons with creamy white scales directed forward, tinged with dark brown scales laterally. Antenna with scape and pecten white, pecten more than 20 bristles; flagellum ochreous white, first segment dark brown above. Labial palpus creamy white; second segment and tuft blackish brown above, mixed with black scales on outer surface and distal half of inner surface; third segment with oblique, blackish brown band near middle. Thorax and tegula creamy white, anterior margin dark brown. Forewing index 0.25, subrectangular, apex bluntly rounded, termen oblique; ground color yellowish white to yellowish brown, shining bluish violet, scattered with unconspicuous, transverse, fine grayish brown striae, with large dark brown spot at distal 3/5 and 2/3 of costa as well as at basal 1/4 near fold; M absent in cell, R_4_ and R_5_ separated; fringe brown. Hindwing index 0.32; pale grayish brown, shining bluish violet, distal 1/5 with faint, grayish brown striae; all veins present, M stem distinct in cell, branched at middle; fringe yellowish brown. Legs creamy white; fore femur with narrow, blackish brown spot on ventral surface, tibia blackish brown on outer surface, first segment of tarsus with large, blackish spot on outer surface, third and fourth segments mixed with dark brown; mid tibia with three broad oblique dark brown bands on outer surface, spurs with oblique dark brown band before apex, first to fourth segments of tarsus with large, blackish brown spot on outer surface; hind tibial tuft pale yellowish brown, forming two pale grayish brown clusters, blackish and shining purple at apex, tarsus and spurs of same pattern with mid leg.

**Male genitalia** (Fig. 15). Corema present; eighth sternite slightly convex at middle on posterior margin. Uncus lobe ovate, heavily sclerotized, with long setae on dorsal surface; apex pointed, setose, with shallow pocket posterolaterally. Subscaphium clubbed, 0.6× length of saccus, anterior 4/5 smooth, posterior 1/5 with elongate setae. Saccus elongate, rodlike, expanded anteriorly. Juxta heavily sclerotized, each lobe rectangular in basal 2/3, scallop-shaped in distal 1/3, with dentation and fine setae on posterior margin. Valva flask-shaped, basal 3/5 about 2.0× width of distal 2/5; process from membrane between valva and juxta papillary, with short setae on posterior margin. Transtilla absent. Labides with anterior 4/5 slender, posterior 1/5 umbrella-shaped. Aedeagus slender, clubbed, 1.4× length of saccus, straight, pointed at apex, complete ventrally and dorsally, with a row of 6 or 7 small spinules.

**Female genitalia** (Fig. 21). Corethrogyne present in seventh segment. Eighth tergite shield-shaped, with sparse short setae, lateral margins slightly concave at anterior 1/3, anterior margin concave at middle, anterolateral side protruding triangularly, posterior margin incised, deeply concave at middle, forming a pair of small papillary lateral processes; sternite tapered, posterior margin concave at middle, forming two overlapped plates, between two plates with a tubular chunnel. Ostium at middle of middle concavity on posterior margin of eighth sternite. Apophysis anterior 0.3× length of apophysis posterior. Antrum heavily sclerotized, divided at posterior 2/3 by a narrow, oblique membranous ring at point of junction with ductus seminalis, posterior 2/3 broader and less sclerotized than anterior 1/3; ductus bursae pipe-shaped, with coarse, transverse, regular constrictions, posterior 4/5 slightly sclerotized, anterior 1/5 slightly expanded and incurvate. Corpus bursae as long as ductus bursae, without signum.

##### Distribution.

China (Fujian, Guangdong, Guangxi).

##### Etymology.

The specific name is derived from the Latin *conchatus* (= conchoidal), referring to the scallop-shaped distal half of the juxta.

#### Tinissa connata sp. n.

urn:lsid:zoobank.org:act:F528F4DB-C10C-470B-A2DA-B95CD21E454B

http://species-id.net/wiki/Tinissa_connata

Figs 1 , 6c , 11 , 16 , 22

##### Type material.

Holotype ♂ − **CHINA**, **Fujian Province:** Guadun (27°44'N, 117°38'E), Mt. Wuyi, 1100 m, 29.VII.2008, leg. Weichun Li, Yongling Sun and Haiyan Bai, genitalia slide No. XYL08114.Paratypes: **Guangdong Province:** 1 ♂, Nanling, Shaoguan City (24°48'N, 113°35'E), 22−28.VI.2008, leg. Liusheng Chen, genitalia slide No. YLL11167. **Guangxi Zhuang Autonomous Region:** 1 ♀, Mt. Daming (23°09'N, 108°16'E), Nanning City, 1200 m, 5.VIII.2011, leg. Shulian Hao and Yinghui Sun, genitalia slide No. YLL11137.

##### Diagnosis.

The new species can be easily recognized by the small-sized body and the rough, raised scales on the first hind tarsal segment. These characters are also present in *Tinissa apicimaculata* sp. n., but the forewing of *Tinissa apicimaculata* sp. n.is darker in color, and has an ovate blackish brown spot near apex that is absent in *Tinissa connata* sp. n.

##### Description.

Adult (Fig. 11): Wingspan 12.5 mm. Vertex and frons ochreous white, tinged with grayish brown around antenna. Antenna with scape and pecten ochreous white, pecten more than 20 bristles; flagellum yellowish brown, first segment with blackish brown above. Labial palpus white, second segment and tuft blackish brown above, mixed with blackish brown on distal half of inner surface and on outer surface, third segment with oblique, blackish brown band before apex. Thorax and tegula white, anterior margin dark brown. Forewing index 0.26, lanceolate, apex protruded triangularly; ground color pale yellowish brown, gradually darker from base to apex, shining bluish violet, with scattered dark brown dots throughout, concentrated from costal 1/4 to dorsal 1/6, forming a discontinuous oblique stria; costal margin with conspicuous creamy white dot at distal 1/3 and 1/4; M with weak trace in cell, R_4_ and R_5_ separated; fringe brown. Hindwing index 0.32; yellowish brown, shining bluish violet; M stem conspicuous in cell, branched at middle; fringe pale yellowish brown. Legs ochreous white; fore coxa and femur mixed with brown on ventral surface, tibia blackish brown ventrally, tarsus with first segment on outer surface as well as fourth and fifth segments blackish brown; mid tibia with blackish brown spot at base, with a narrow, blackish brown band at middle, with an oblique dark brown band before apex, spurs with oblique dark band before apex, first segment of tarsus with blackish brown spot at base and middle, fourth segment dark brown; hind tibial tuft pale yellowish brown, distal scales with dark brown tips, tarsus with first segment dark brown on distal half, bearing rough, raised scales on dorsal surface, second segment with small blackish brown dot near base dorsally, fourth segment dark brown.

**Male genitalia** (Fig. 16). Corema present; eighth sternite straight on posterior margin. Uncus lobe deeply emarginated posteriorly, forming two processes: inner process short, bears stub spiculas; outer process 2.5× length of inner one, with spiculas varying in length, pectinated; with a large rounded flap arising from near posterior margin. Subscaphium cheliform, anterior 3/4 slender, slightly curved ventrad, posterior 1/3 broadened, with deeply U-shaped concavity at middle on posterior margin. Saccus broad triangular, narrowed anteriorly, rounded apically. Juxta sclerotized, each lobe triangular, pointed at apex, serrate along inner margin. Valva fully fused with juxta; process from membrane between valva and juxta foliole-shaped, bearing a small apical process and some short setae before apex. Transtilla trapezoidal, fully fused with labides. Labides peach-shaped. Aedeagus stout, as long as saccus, broadened to flared apex, slightly curved ventrad, ventrally complete, with large, triangular flap at base, dorsally with a deep cleft, without carina or spinule.

**Female genitalia** (Fig. 22). Corethrogyne absent, with deciduous setae on posterior margin of seventh segment. Eighth tergite semicircularly concave on anterior margin, and forming rounded plate posterolaterally, with a few short setae on posterior margin; eighth sternite semicircularly concave on anterior margin, forming semi-cylindrical plate anterolaterally extending to tergite, attached with apophysis posterior, setose and slightly concave at middle on posterior margin, with ovate ventral protuberance on ventral margin of ostium. Apophysis anterior 0.3× length of apophysis posterior. Antrum short, divided at posterior 2/3 by a membranous ring at point of junction with ductus seminalis on right; ductus bursae 3.0× length of corpus bursae, posterior 1/5 with transverse constrictions, anterior 4/5 smooth. Corpus bursae pear-shaped, with large, heavily sclerotized, inverted funnel-shaped signum.

##### Distribution.

China (Fujian, Guangdong, Guangxi).

##### Etymology.

The specific name is derived from the Latin *connatus* (= connate), referring to the fusion of the juxta and valva.

##### Remarks.

The single female specimen was not collected in the same locality as the male specimens, but it has the same forewing patterns as the males do, and its first hind tarsal segment has the rough, raised scales, which are characteristic for this species. The rough, raised scales on the first hind tarsal segment are also present in *Tinissa apicimaculata* sp. n. and *Tinissa spaniastra* Meyrick, 1932, but the forewing of *Tinissa apicimaculata* sp. n. has an ovate blackish brown spot near apex, and the members of *Tinissa spaniastra* are larger in size (25 mm in male, 20–27 mm in female (Robinson 1976)). In addition, the genitalia structures of the above mentioned species are also different.

#### Tinissa leguminella sp. n.

urn:lsid:zoobank.org:act:D9425924-508D-475F-B2A6-A21B746E84DC

http://species-id.net/wiki/Tinissa_leguminella

Figs 1 , 12 , 17

##### Type material.

Holotype ♂ − **CHINA**, **Yunnan Province:** Rare Botanical Garden, Ruili (24°00'N, 97°50'E), 1000 m, 5.VIII.2005, leg. Yingdang Ren, genitalia slide No. YLL11139.Paratype: ♂, same data as holotype except dated 7.VIII.2005, genitalia slide No. XYL05048.

##### Diagnosis.

*Tinissa leguminella* sp. n. is similar to *Tinissa indica* in having a similar forewing pattern, a broad and triangular saccus and a short and conical valva in the male genitalia. However, the new speciescan be recognized from the latter by the beanpod-shaped uncus lobe, the bifurcate subscaphium, the horn-shaped juxta, the process from the membrane between the valva and the juxta with basal 3/5 nearly parallel dorso-ventrally, widended at distal 2/5, then narrowed to melanised and setose apex, and the aedeagus with short carina in the male genitalia. In *Tinissa indica*, the uncus lobe is crescent, the subscaphium is triangular, the juxta is fist-shaped, the process from the membrane between the valva and the juxta is fingerlike, and the aedeagus has long carina in the male genitalia.

##### Description.

Adult (Fig. 12): Male wingspan 16.5−19.0 mm. Vertex ochreous yellow, tinged with blackish brown near eyes; frons ochreous yellow, with blackish brown scales laterally. Antenna with scape and pecten ochreous white, pecten more than 20 bristles; flagellum yellowish brown, first two segments blackish brown above. Labial palpus creamy white; second segment brown on outer surface, mixed with creamy white at middle and apex, tuft black; third segment with dark brown spot at base and distal 1/3 on outer surface. Thorax ochreous white, posterior 1/3 grayish brown; tegula creamy white, anterior 1/3 dark brown, posterior 1/3 mixed with yellowish brown. Forewing index 0.25, rectangular, apex protruded triangularly, termen slightly concave inward at about anterior 1/3; ground color brown, shining dark purplish, scattered with conspicuous white spots throughout, regularly arranged along margins as well as between veins, more white spots concentrated in basal 1/5, near fold and at upper angle of cell; M absent in cell, R_4_ and R_5_ separated; fringe brown. Hindwing index 0.32; pale grayish brown, shining dark purplish, with small pale dots apically; M stem conspicuous in cell, branched at middle; fringe yellowish brown. Fore leg yellowish brown, femur with narrow, dark brown spot on ventral surface, tibia blackish brown, tarsus blackish brown except apex of first segment as well as fifth segment ochreous white; mid leg ochreous yellow, tibia with three oblique, blackish brown bands on outer surface, broader near apex, shorter spur with oblique blackish brown band on outer surface, longer spur yellowish brown on outer surface, tarsus with first segment dark brown at base and middle, third and fourth segments dark brown; hind leg pale yellowish brown, tibia ochreous white at basal 2/3 ventrally, tuft dark grayish brown, forming two clusters at spurs, spurs ochreous white ventrally, dark brown dorsally but yellowish at apex, first segment of tarsus ochreous white ventrally, with dark brown spot at base on outer surface, with large, dark brown spot from basal 1/3 to before apex on outer surface, other tarsal segments yellowish brown ventrally, third and fourth segments blackish brown dorsally.

**Male genitalia** (Fig. 17). Corema present; eighth sternite straight on posterior margin. Uncus lobe beanpod-shaped, hornlike and heavily sclerotized, sparsely setose on distal half, with shallow pocket distally. Subscaphium fused anteriorly, bifurcate from 1/4, forming long band-shaped lobe on each side, gradually narrowed to blunt apex. Saccus broad triangular. Juxta heavily sclerotized, each lobe stout, narrow basally, dilated distally; apex straight, setose, and melanised; basally fused and protruded ventrad, forming a plate with a vertical ridge at middle. Valva short, conical, apex narrowly rounded, with long distal setae; process from membrane between valva and juxta with basal 3/5 nearly parallel dorso-ventrally, widended at distal 2/5, then narrowed to melanised and setose apex. Transtilla broad, inverted peach-shaped. Labides concave at middle on posterior margin, with mastoid process posterolaterally. Aedeagus stout, clubbed, 1.5× length of saccus, gently curved dorsad, complete dorsally and ventrally, with a short carina arising from distal 1/4 ventrally.

**Female.** Unknown.

##### Distribution.

China (Yunnan).

##### Etymology.

The specific name is derived from the Latin *legumin*- (= legume) and the postfix -*ellus*, referring to the beanpod-shaped uncus lobe.

#### Tinissa spirella sp. n.

urn:lsid:zoobank.org:act:151565EB-709E-4628-AAD4-81CCDE40A961

http://species-id.net/wiki/Tinissa_spirella

Figs 1 , 13 , 18

##### Type material:

Holotype ♂ − **CHINA**, **Sichuan Province:** Wolong Nature Reserves (31°01'N, 103°10'E), 1900 m, 7.VIII.2004, leg. Yingdang Ren, genitalia slide No. XYL05050.

##### Diagnosis.

The new species is similar to *Tinissa conchata*sp. n. by having an elongate, rodlike saccus and a slender aedeagus. It can be recognized from the latter by the L-shaped uncus lobe with an apical thorn, the subovate subscaphium with slender fingerlike process on each side, the spiral juxta, the pyramidical valva, and the needlelike aedeagus curved dorsad, without spinule. In *Tinissa conchata*sp. n., the uncus lobe is ovate, with shallow pocket posterolaterally; the subscaphium is clubbed, with elongate setae posteriorly, the juxta is rectangular in basal 2/3 and scallop-shaped in distal 1/3, the valva is flask-shaped, and the clubbed aedeagus is straight, with a row of small spinules.

##### Description.

Adult (Fig. 13): Male wingspan 20.0 mm. Vertex ochreous yellow, posterior margin ochreous white; frons ochreous white, tinged with ochreous yellow. Antenna with scape and pecten ochreous white, pecten about 10−20 bristles; flagellum pale yellowish brown, first segment blackish brown above. Labial palpus with first and second segments creamy white on inner surface, dark brown mixed yellowish brown on outer surface; third segment ochreous yellow, with yellowish brown spot at base and before apex on outer surface. Thorax creamy white mixed with dark grayish brown scales; tegula with anterior half dark grayish brown, posterior half creamy white but pale gray at middle. Forewing index 0.27, rectangular, apex protruded triangularly, termen slightly concave inward at about 2/5; yellowish brown mixed with grayish brown, shining bluish violet, with scattered faint white dots, large and conspicuous at base, along costa, termen and dorsum; M absent in cell, R_4_ and R_5_ separated. Hindwing index 0.32; grayish brown, shining bluish violet; M stem conspicuous in cell, branched at middle; fringe pale grayish brown. Legs yellowish brown; fore femur dark brown on ventral surface, tarsus with first segment blackish brown on outer surface, with faint dark brown spots on outer surface of second to fifth segments; mid tibia with blackish brown spot at base, with one narrow, blackish brown band at middle, with one broad, oblique, dark brown band before apex on outer surface, tarsus with small, blackish brown spot at base and middle on outer surface of first segment; hind tibial tuft pale yellowish brown, blackish brown before apex on outer surface, tarsus with first segment dark brown at base and apex on dorsal surface, with long, fine scales dorsally, third segment dark brown dorsally.

**Male genitalia** (Fig. 18). Corema present; eighth sternite straight on posterior margin. Uncus lobe L-shaped, widely spaced to each other, completely fused with vinculum; distal half setose dorsally, with a large apical thorn. Subscaphium bulletlike, with a pair of small and narrow triangular protuberances at middle on anterior margin, with a slender fingerlike process arising from anterior 2/5 of each side, 0.5× length of subscaphium. Saccus rodlike. Juxta heavily sclerotized, each lobe spiral, with long setae ventrally, fused on inner margin. Valva short, heavily sclerotized, more or less triangular, apex pointed, dorsal margin with long setae; process from membrane between valva and juxta mastoid, with short setae at apex. Transtilla absent. Labides small, triangular, heavily sclerotized. Aedeagus very slender, needlelike, 1.2× length of saccus, curved dorsad, complete dorsally and ventrally, roundly protruded at base ventrally, without carina or spinule.

**Female.** Unknown.

##### Distribution.

China (Sichuan).

##### Etymology.

The specific name is derived from the Latin *spirellus* (= spiral), referring to the small, spiral, whorl-shaped juxta.

## Discussion

The genus *Tinissa* is unique among the 30 genera of the subfamily Scardiinae. It is highly diagnostic by the hind legs bearing large tufts of erect scales at apex of the tibia. Based on this character, we assign five new species to this genus.

As all the nominal species were well described and illustrated by previous taxonomists (Robinson 1976, 1981; Robinson and Tuck 1998; Gozmány and Vári 1973; Zagulajev 1972), we examined only the holotypes of *Tinissa araucariae* Robinson, 1976, *Tinissa bakeri* Robinson, 1976, *Tinissa insignis* Zagulajev, 1972, *Tinissa parallela* Robinson, 1976, *Tinissa polysema* Zagulajev, 1972) as well as the paratype of *Tinissa insularia* Robinson, 1976 that were available during this study. Besides, the shape of valva+juxta complex and the uncus lobes in the male genitalia as well as the eighth sternite and the ostium in the female genitalia are highly diverse among species, which makes the species identification more effective.

## Supplementary Material

XML Treatment for Tinissa

XML Treatment for Tinissa indica

XML Treatment for Tinissa insularia

XML Treatment for Tinissa apicimaculata

XML Treatment for Tinissa conchata

XML Treatment for Tinissa connata

XML Treatment for Tinissa leguminella

XML Treatment for Tinissa spirella
